# Taeniasis among Refugees Living on Thailand–Myanmar Border, 2012

**DOI:** 10.3201/eid2110.141657

**Published:** 2015-10

**Authors:** Ellen J. McCleery, Prapas Patchanee, Pornsawan Pongsopawijit, Sasisophin Chailangkarn, Saruda Tiwananthagorn, Papaspong Jongchansittoe, Anchalee Dantrakool, Nimit Morakote, Hnin Phyu, Patricia P. Wilkins, John C. Noh, Christina Phares, Seth O’Neal

**Affiliations:** Oregon Health & Science University, Portland, Oregon, USA (E.J. McCleery, S. O’Neal);; Chiang Mai University, Chiang Mai, Thailand (P. Patchanee, P. Pongsopawijit, S. Chailangkarn, S. Tiwananthagorn, A. Dantrakool, N. Morakote);; Thailand Division of Livestock Development, Mae Hong Son, Thailand (P. Jongchansittoe);; International Rescue Committee, Mae Hong Son (H. Phyu);; Centers for Disease Control and Prevention, Atlanta, Georgia, USA (P.P. Wilkins, J.C. Noh, C. Phares)

**Keywords:** taeniasis, cysticercosis, Taenia solium, refugees, Thailand, Myanmar, epidemiology, parasites

## Abstract

We tested refugee camp residents on the Thailand–Myanmar border for *Taenia solium* infection. Taeniasis prevalence was consistent with that for other disease-endemic regions, but seropositivity indicating *T. solium* taeniasis was rare. Seropositivity indicating cysticercosis was 5.5% in humans, and 3.2% in pigs. Corralling pigs and providing latrines may control transmission of these tapeworms within this camp.

Infection with the adult form of *Taenia solium* tapeworms, known as taeniasis, results from consuming undercooked or raw pork that is contaminated by this parasite (http://www.cdc.gov/parasites/taeniasis/gen_info/faqs.html). Though these infections are often asymptomatic, infection with tapeworm eggs can progress to a larval infection of the central nervous system, known as neurocysticercosis, that poses a serious public health hazard. Neurocysticercosis is a major cause of acquired epilepsy in the developing world (*1**–**3*) and an emerging public health issue in the United States because of emigration from and travel to areas in Latin America, Asia, and Africa where the disease is endemic (*4**–**6*). Multiple cases of neurocysticercosis have been reported among resettled refugees from Myanmar, one of the largest refugee groups recently resettled in the United States (*7**–**10*). A recent seroprevalence survey showed that 1 in 4 refugees from Myanmar has antibodies against *T. solium* cysts (*8*), which suggests that *T. solium* infection might be endemic to Myanmar or camps in Thailand where refugees reside before they are resettled. However, because little is known about transmission of this zoonotic parasite in that region, opportunities for disease control and prevention have been limited.

## The Study

In October 2012, we conducted a cross-sectional study in the Ban Mai Nai Soi refugee camp on the Thailand–Myanmar border. This camp was established in 1996 to house persons displaced by conflicts between ethnic minorities and the Myanmar government. Many of these refugees subsequently resettle in the United States. Approximately 13,591 persons in roughly 3,000 households lived in the camp as of October 2012 (*11*). Camp residents live in closely packed bamboo housing; many have small yards with an enclosed pit latrine. Residents do not have access to electricity and obtain treated water from communal water stations located throughout the camp. Domestic pig rearing is common, and corral use is mandated by camp authorities.

We randomly selected participants from all houses in the camp by using hand-drawn maps provided by the camp’s governing committee and invited all household residents, regardless of age, to participate. We interviewed all consenting residents using a standard questionnaire in Karenni, their primary language, and collected a fecal sample and blood sample from them. We attempted to interview all participants directly, but allowed parents to answer questions for their young children. We examined whole fecal samples macroscopically in a field laboratory for *Taenia* sp. proglottids or scoleces (i.e., segments or anterior ends) but did not use morphologic characteristics to identify the species of the recovered tapeworm material. Fecal and blood samples were transported on ice each day to the laboratory at Chiang Mai University for further processing. Fecal samples were concentrated by sedimentation and examined by light microscopy for *Taenia* sp. eggs. Pig blood was analyzed by enzyme-linked immunoelectrotransfer blot (EITB) for antibodies against *T. solium* cysticerci (*12*). Human blood was analyzed by EITB for antibodies against recombinant antigens specific to *T. solium* tapeworms (rES33) and cysticerci (rT24) (*12**,**13*). The sensitivity and specificity of these tests are 97% and 100%, respectively, for rES33, and 94% and 98%, respectively, for rT24. Participants with taeniasis were given a single oral dose of niclosamide with bisacodyl to assist in tapeworm elimination (additional methods are provided in the Technical Appendix.

We interviewed 738 persons in 205 randomly sampled households, representing roughly 5% (738/13,591) of the total camp population (Table). Our sample included a higher proportion of girls and women (394, 53.4%), and participants’ mean age was 23.7 years (SD ± 19.8), ranging from a few months to 84 years of age. Participants had a mean of 2.8 years of education (SD ± 3.6) and had lived in the camp for an average of 9.9 years (SD ± 5.9). Most participants were unemployed (575, 80.0%) and owned at least 1 pig (549, 74.4%); none owned a cow. Among the 138 (67%) households in which pigs were raised, all but 1 (≈100%) household maintained their pigs in a corral. Approximately one quarter of participants reported eating raw pork (190, 26.1%) and raw beef (178, 24.6%).

**Table:** Characteristics of refugees living on the Thailand–Myanmar border who participated in taeniasis study and their households, 2012*

Characteristic	Finding
Refugees, n = 738	
Age, y, median (IQR); mean (SD)	18 (7–35); 23.7 (19.8)
Education, y, median (IQR); mean (SD)	1 (0–5); 2.8 (3.6)
Lived in camp, y, median (IQR); mean (SD)	11 (4–16); 9.9 (5.9)
Female sex, no. (%)	394 (53.4)
Unemployed, no. (%)	575 (80.0)
Households, n = 205	
No. residents, median (IQR); mean (SD)	4 (4–6); 4.3 (1.9)
Main floor elevated, no. (%)	169 (83.7)
Main floor cement or bamboo, no. (%)	198 (98.5)
Latrine in yard, no. (%)	170 (83.7)
Water runs from tap to house, no. (%)	35 (17.2)
At least 1 pig, no. (%)	138 (67.3)

*IQR, interquartile range.

Fecal samples were collected from 552 (75%) participants. Of these, 18 (3.3%) participants tested positive for taeniasis (i.e., eggs or proglottids were detected by microscopy), ranging from 1 (0.6%) person <10 years of age to 7 (11.7%) persons >54 years of age (Figure). After accounting for intrahousehold clustering and sampling weight, the prevalence of taeniasis as determined by microscopy was 2.9% (95% CI 1.4%–4.3%). Among the 671 persons with blood sample results, only 1 (0.1%) had serologic test results indicating *T. solium* taeniasis. This person was negative for *Taenia* sp. eggs or proglottids via fecal microscopy and was not seropositive for antibodies indicating *T. solium* cysticercosis.

**Figure:**
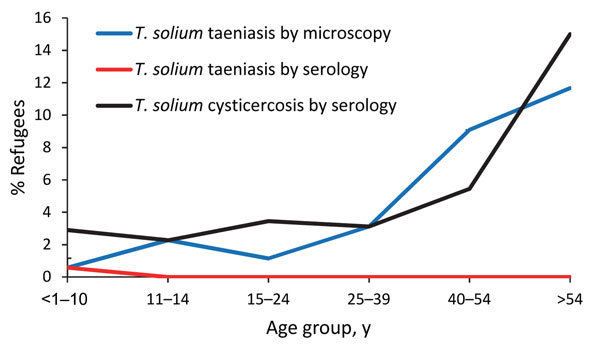
*Taenia solium* taeniasis and cysticercosis in refugees living on the Thailand–Myanmar border, 2012. Taeniasis by microscopy indicates presence of *T. solium* eggs or proglottids in stool.

Blood samples were collected from 671 (91%) participants. Of these, 29 (4.3%) were seropositive for antibodies against *T. solium* cysticerci by using EITB rT24, ranging from 6 (2.9%) persons <10 years old to 10 (15%) persons >54 years old (Figure). After accounting for household clustering and sampling weight, seroprevalence was 5.5% (95% CI 2.9%–8.1%). Of the 258 pigs whose blood we collected, 11 (4.3%) were seropositive for antibodies against *T. solium* cysticerci by EITB lentil-lectin purified glycoprotein. After adjustment for household clustering and sampling weight, 3.2% (95% CI 0.8%–5.5%) were seropositive.

## Conclusions

Taeniasis is relatively common among residents of the Ban Mai Nai Soi refugee camp. The overall prevalence of 2.9% is consistent with estimates from other regions where *Taenia* sp. are endemic. Although we did not definitively identify the species of taeniasis present, serologic results suggest that *T. solium* is not the dominant *Taenia* species in this population. We found only 1 person with serum antibodies indicating *T. solium* taeniasis using an assay that is 100% specific for *T. solium*. The prevalence of antibodies indicating *T. solium* cysticercosis in humans was also low. Given this combination of findings, *T. asiatica* or *T. saginata* are likely the dominant species in this camp. The risk of acquiring neurocysticercosis in Ban Mai Nai Soi is therefore likely to be relatively low. However, the distribution of *Taenia* sp. tapeworms varies considerably among geographic areas and ethnic groups, so these results might not be generalizable to other camps or communities along the Thailand–Myanmar border.

The universal availability of sanitary infrastructure and wholesale adoption of animal corralling within the camp limit the conditions necessary for the *T. solium* tapeworm to complete its lifecycle. These prevention measures might control transmission despite the pervasive poverty and common practice of eating raw pork. Sanitation and pig restraint within the camps and surrounding communities might reduce the risk for *T. solium* taeniasis and neurocysticercosis in this region.

This study has limitations. Because we collected only 1 fecal sample from each participant, we might have underestimated the prevalence of taeniasis. Although we recovered tapeworm material from fecal samples, we were not able to perform any molecular analyses, so the exact species of *Taenia* remains unconfirmed. Many of the variables collected were self-reported and therefore subject to participants’ recall bias.

Additional epidemiologic studies are needed to improve understanding of the distribution of *Taenia* sp. infections in this region and the effects of associated neurologic disease. Screening and treatment for taeniasis among refugees before resettlement might also reduce the risk of further transmission in the receiving country.

Technical Appendix Detailed methods for study of *Taenia solium* taeniasis and cysticercosis in refugees living on the Thailand–Myanmar border, 2012.

## References

[1] Coyle CM, Mahanty S, Zunt JR, Wallin MT, Cantey PT, White AC Jr, Neurocysticercosis: neglected but not forgotten. PLoS Negl Trop Dis. 2012;6:e1500. 10.1371/journal.pntd.0001500 22666505 PMC3362619

[2] Garcia HH, Del Brutto OH. Neurocysticercosis: updated concepts about an old disease. Lancet Neurol. 2005;4:653–61. 10.1016/S1474-4422(05)70194-0 16168934

[3] García HH, Gonzalez AE, Evans CA, Gilman RH; Cysticercosis Working Group in Peru. *Taenia solium* cysticercosis. Lancet. 2003;362:547–56. 10.1016/S0140-6736(03)14117-7 12932389 PMC3103219

[4] Del Brutto OH. Neurocysticercosis among international travelers to disease-endemic areas. J Travel Med. 2012;19:112–7. 10.1111/j.1708-8305.2011.00592.x 22414036

[5] Jongwutiwes U, Yanagida T, Ito A, Kline SE. Isolated intradural-extramedullary spinal cysticercosis: a case report. J Travel Med. 2011;18:284–7. 10.1111/j.1708-8305.2011.00535.x 21722242

[6] O’Neal SE, Robbins NM, Townes JM. Neurocysticercosis among resettled refugees from Burma. J Travel Med. 2012;19:118–21. 10.1111/j.1708-8305.2011.00588.x 22414037

[7] Hewagama SS, Darby JD, Sheorey H, Daffy JR. Seizures related to praziquantel therapy in neurocysticercosis. Med J Aust. 2010;193:246–7. 20712550 10.5694/j.1326-5377.2010.tb03885.x

[8] O’Neal SE, Townes JM, Wilkins PP, Noh JC, Lee D, Rodriguez S, Seroprevalence of antibodies against *Taenia solium* cysticerci among refugees resettled in United States. Emerg Infect Dis. 2012;18:431–8. 10.3201/eid1803.111367 22377408 PMC3309588

[9] Pluschke M, Bennett G. Orbital cysticercosis. Aust N Z J Ophthalmol. 1998;26:333–6. 10.1111/j.1442-9071.1998.tb01339.x 9843263

[10] Yeaney GA, Kolar BS, Silberstein HJ, Wang HZ. Case 163: solitary neurocysticercosis. Radiology. 2010;257:581–5. 10.1148/radiol.10090856 20959550

[11] The Border Consortium. Refugee and IDP camp populations: October 2012 [cited 2015 Aug 3]. http://www.theborderconsortium.org/media/11741/2012-10-oct-map-tbbc-unhcr-1-.pdf

[12] Hancock K, Pattabhi S, Whitfield FW, Yushak ML, Lane WS, Garcia HH, Characterization and cloning of T24, a *Taenia solium* antigen diagnostic for cysticercosis. Mol Biochem Parasitol. 2006;147:109–17. 10.1016/j.molbiopara.2006.02.004 16540186

[13] Levine MZ, Lewis MM, Rodriquez S, Jimenez JA, Khan A, Lin S, Development of an enzyme-linked immunoelectrotransfer blot (EITB) assay using two baculovirus expressed recombinant antigens for diagnosis of *Taenia solium* taeniasis. J Parasitol. 2007;93:409–17. 10.1645/GE-938R.1 17539427

