# Supplementary material for: Taeniasis among Refugees Living on Thailand–Myanmar Border, 2012
Source: Emerg Infect Dis. 2015 Oct;21(10):1824–6. doi: 10.3201/eid2110.141657 (PMC4593425; doi:10.3201/eid2110.141657)
Supplement: Technical Appendix — Detailed methods for study of Taenia solium taeniasis and cysticercosis in refugees living on the Thailand–Myanmar border, 2012. [file 14-1657-Techapp-s1.pdf]

Article DOI: http://dx.doi.org/10.3201/eid2110.141657

Taeniasis among Refugees Living on the Thailand-Myanmar Border, 2012

Technical Appendix

Methods

Specimen Collection

We asked all participants to provide a whole stool sample. Each participant was provided a 500-mL plastic container and a bar of soap and was instructed on hygienic collection of stool. Field teams returned within 24 hours to collect stool samples. We also collected a blood sample via fingerstick. Lab technicians used disposable lancets and semi-quantifiable filter paper strips (Nobuto, Tokyo, Japan) to collect ≈1 mL of blood per participant. Used filter paper strips were stored in microtubules containing stabilizing enzyme (Stabilzyme SELECT, SurModies, Eden Prairie, MN, USA).

Veterinarians collected a blood sample from all pigs by using disposable lancets and filter paper strips to collect ≈1 mL of blood from the inside of the pig’s ear flap. Used filter paper strips were stored in microtubules containing stabilizing enzyme.

Statistical Analyses

All statistical analyses were performed with Stata version 12.1 (Stata Statistical Software: Release 12. StataCorp LP, College Station, TX, USA). The crude prevalence of human taeniasis and seroprevalence of antibodies against cysticercosis were calculated as the proportion of persons with corresponding positive laboratory results among all persons sampled. Crude seroprevalence or porcine cysticercosis was calculated as the proportion of pigs with positive laboratory results among all pigs sampled. To adjust for intrahousehold clustering of participants, we assigned the household identification number as the panel variable on all models. We calculated sampling weights for each participant by dividing the total number of households in the camp (n = 2,988) by the number of households surveyed (n = 205) and then multiplying by the reciprocal of the

participant’s number of household members. We calculated 95% CIs for all prevalence estimates.

Human Subjects Protections and Animal Welfare

We obtained verbal consent from every participant ≥18 years of age. An assent script was used with children <18 years of age, and a parent or guardian was also required to provide consent for the minor to participate. The Institutional Review Boards at Oregon Health & Science University and Chiang Mai University Faculty of Medicine as well as the Institutional Animal Care and Use Committees at Oregon Health & Science University and Chiang Mai University Faculty of Veterinary Medicine, approved this research.

Financial Support

This research was made possible with financial support from the Oregon Clinical and Translational Research Institute, grant no. 5KL2 RR024141-04 from the National Center for Advancing Translational Sciences, a component of the National Institutes of Health, and the National Institutes of Health Roadmap for Medical Research.
